# Programmable material testing device for mechanoluminescence measurements

**DOI:** 10.1016/j.ohx.2022.e00349

**Published:** 2022-08-24

**Authors:** Ernests Einbergs, Aleksejs Zolotarjovs

**Affiliations:** Institute of Solid State Physics, University of Latvia, Riga LV-1063, Latvia

**Keywords:** Mechanoluminescence, Material testing system, Strain, Tensile test, Three-point flexural test, 3D printing

## Abstract

Mechanoluminescent materials transform mechanical energy into visible light. Phenomena could prove to be advantageous to various next-generation monitoring systems employed in the fields of security and healthcare if the intrinsic mechanisms are fully understood. Scientific efforts are mainly hindered by the lack of equipment capable of controlled mechanical deformation and simultaneous collection of light emitted by the sample. This article describes an easily constructible material testing device (508 €) with an interchangeable test fixture and an integrated load cell made from readily available mechanical components and 3D printed parts. A commercial low-cost alternative to spectroscopic apparatus (200 €) has recently become available alongside a highly capable 16-bit CMOS camera intended for low light conditions (520 €). A highly modular prototype system with an overall cost much lower than commercial alternatives that provide less functionality could enable a larger portion of scientific personnel to contribute to a novel field of research.

## Graphical abstract

**Figure:**
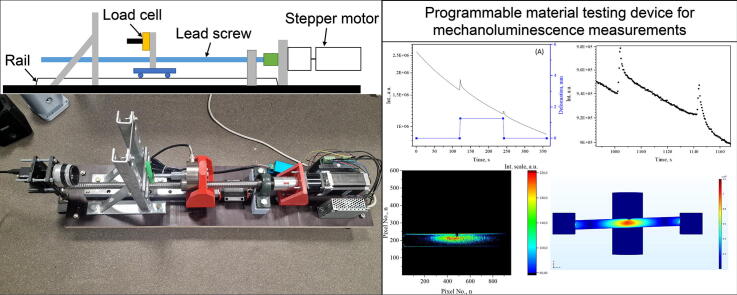

## Specifications table

**Table:** 

Hardware name	Programmable material testing device for mechanoluminescence measurements
Subject area	• Engineering and materials science • General
Hardware type	• Imaging tools • Measuring physical properties and in-lab sensors • Mechanical engineering and materials science
Closest commercial analog	Computer-controlled electronic universal testing machine
Open-source license	CC BY 4.0
Cost of hardware	1216.52 €
Source file repository	All supporting software is available at https://DOI.org/10.17605/OSF.IO/S4PG7

## Hardware in context

The discovery of elastic-luminescence (EML) attracted the attention of a considerable amount of researchers. Non-destructive luminescence-based deformation sensor materials could form the backbone of a variety of optoelectric sensors and displays. Stress field visualization in an in-situ manner provides a previously incomprehensible opportunity to optimize the structural integrity and geometrical shape of various mechanical components and structural elements with a quick measurement under load. Since EML materials can be excited by electric [1] or magnetic [2] fields when properly coupled with piezoelectric crystals, a novel security system can be created that is sensitive not only to mechanical deformation but also to residual light, change in electric/magnetic field due to the presence of an absorbent object and in some cases to the change in ambient temperature [3].

Despite the many advantages this group of materials could provide in the fields of security and healthcare, most mechanoluminescent phosphors have been discovered in a trial-and-error approach and most of the currently well-known materials are not commercialized due to the limited range of colors and limited sensitivity, and intensity. Publications tend to attribute the lack of purposeful studies to the absence of an all-encompassing theory that could explain the variety of observed phenomena when dealing with EML materials [4]. A considerable part of the general scientific personnel is incapable of contributing to the research of mechanoluminescent (ML) materials for the most part due to the lack of commercial measurement systems.

A setup that is capable of deformation and the collection of the emitted light is required. Such a system must be deterministic - precise measurement of displacement, the load applied to the sample as well as the sensitivity to light should be well defined or measured. Material testing devices are expensive, hardly modifiable, and often do not include the required equipment to acquire images or spectra from the sample being tested. Scientific institutions with the required light measurement systems typically do not own a material testing device and vice versa.

The development of an easily reproducible measurement system will serve as a gateway to a more diverse scientific field. Proven and tested hardware with accompanying software could persuade scientists that were previously daunted by such an undertaking to adapt the available resources and contribute to a large barren field of novel research. The prototype design described in this publication is intended to serve as inspiration to a scientific institution that already possesses one or multiple of the major components required in the construction of such a system which would curve the total costs. Two of the most expensive components of the whole system are spectroscopic devices and image acquisition equipment, both are widely available to scientists researching the field of luminescence. The use of 3D printed parts considerably reduces overall expenses which would arise from the machining of geometrically complex parts and is generally faster and more sustainable as the replacement of parts is easy.

## Hardware description

The device was devised with simplicity and modularity in mind. All parts are interchangeable and adaptable to specific needs. The device is divided into three modules. The first module is responsible for force generation and movement. The second module holds the sample and determines the type of deformation. The third module is comprised of equipment that registers luminescence emitted from samples during deformation.

### Module one – force application device

**Fig. 1:**
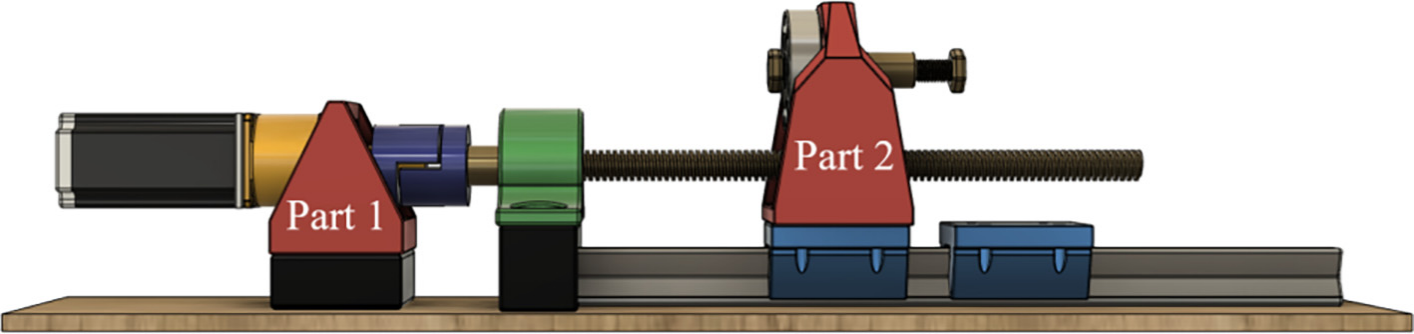
Module one – force application device.

The first module consists of a rail and cart system mounted to a base plate and a stepper motor which rotates the ball screw through the gearbox (Fig. 1). The ball screw nut transfers the rotational motion of the ball screw to lateral movement by being mounted to a support structure seated on a cart (Part 2). To reduce the intrinsic strain on the system due to misalignment or large torque, a stepper motor is connected to its planetary gear set and mounted to another supporting structure (Part 1). The design of Part 2 mostly depends on the shape and design of the load cell chosen; in this case, a round uniaxial load cell was chosen. Cut-outs are lead screw and mounting screw dependent. Force exerted upon motor housing structure (Part 1) is close to that related to the combined weight of the motor itself and torque that is needed to rotate the ball screw and it is not required for it to be overwhelmingly structurally sound. The motor is interchangeable.

### Module two

**Fig. 2:**
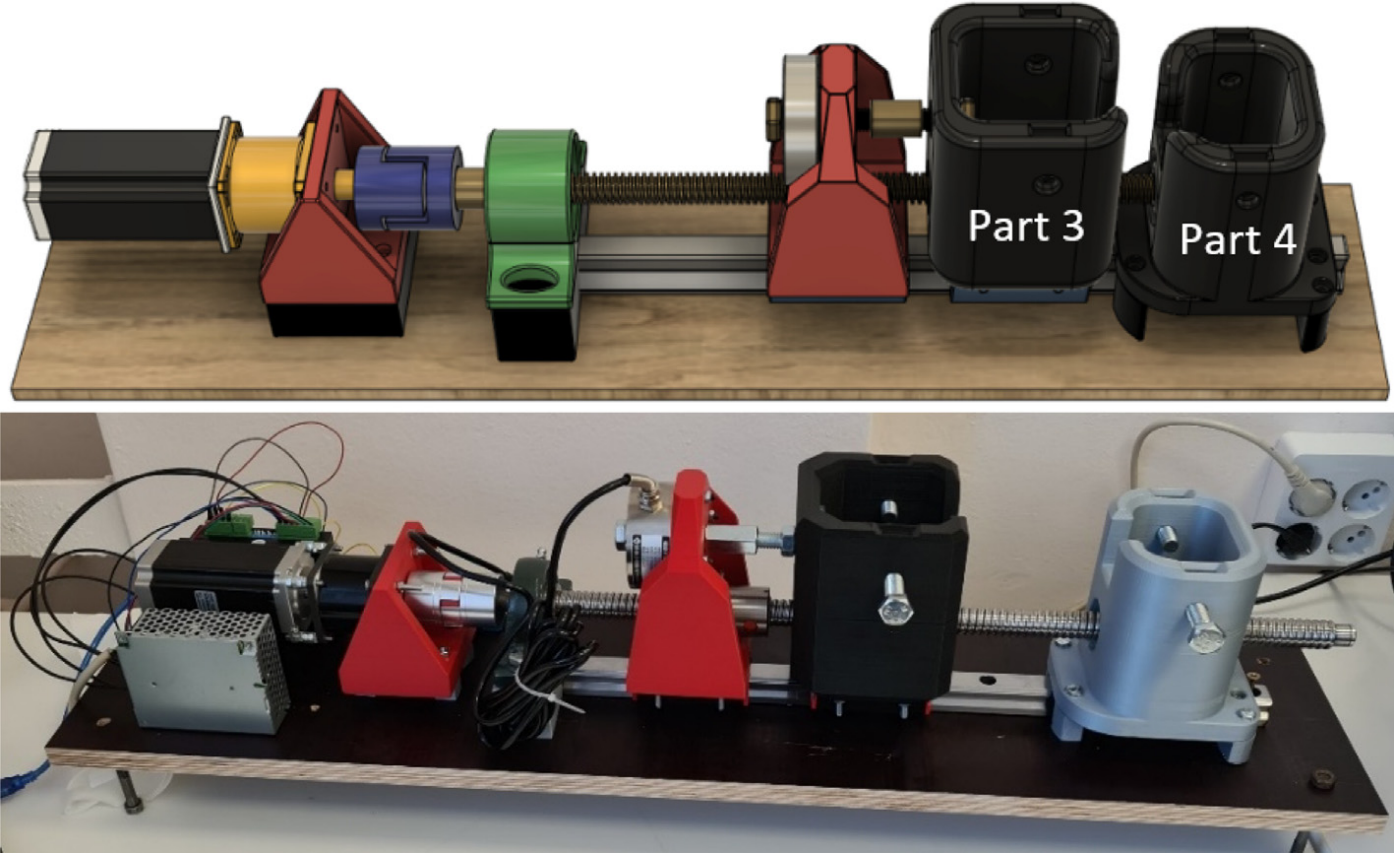
Material testing device in tensile testing configuration.

**Fig. 3:**
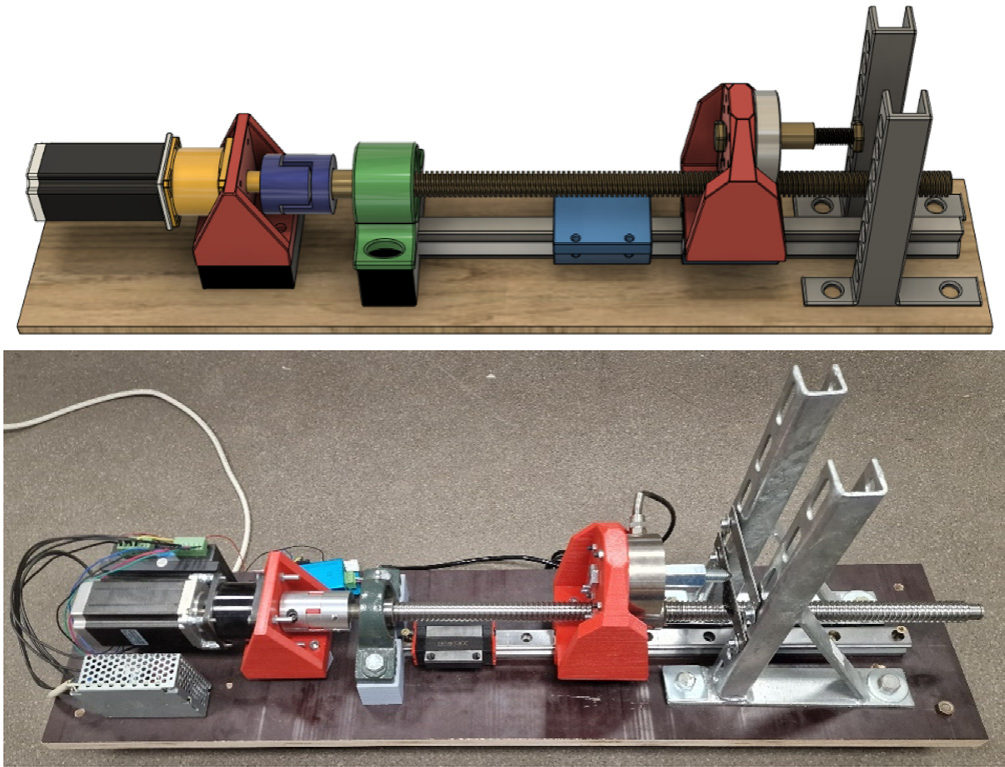
Material testing device in the three-point flexural test configuration.

This module determines the type of deformation the sample will be subjected to. A tested prototype for performing mechanoluminescence measurements during tensile testing and three-point flexural tests is provided. Visualization and execution of tensile testing configuration can be observed in Fig. 2 and the three-point flexural test in Fig. 3. Only tensile testing configuration requires 2 carts. Parts 3 and 4 have 2 slits for an M12 nut which provides threading for the clamping screws and will not wear out after multiple samples.

### Module three

**Fig. 4:**
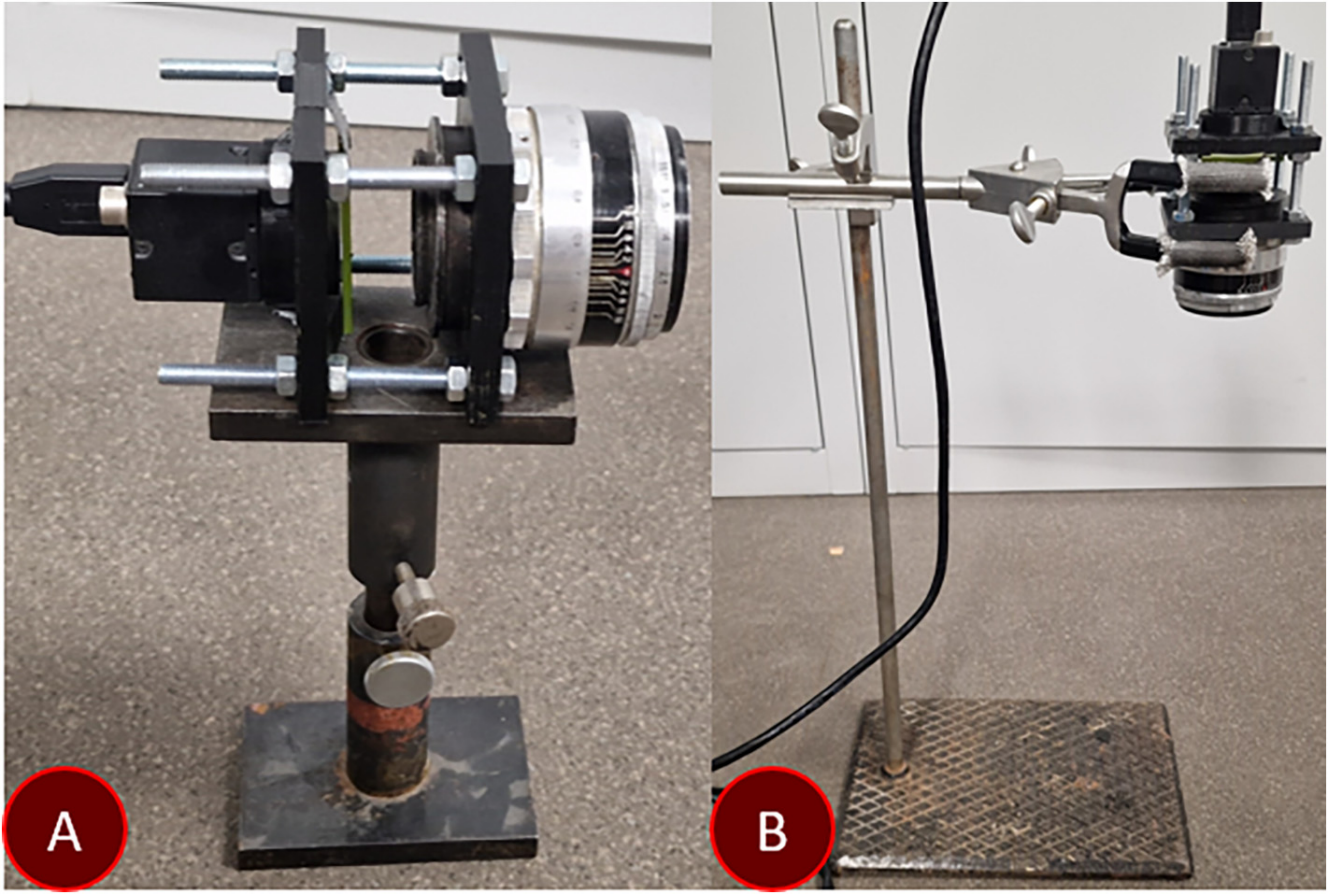
(a) Camara module on a height adjustable table and (b) in a finger clamp holder. Images of a fully assembled device with the CMOS camera module are shown in Fig. 5 and Fig. 6.

The measurement module determines the overall error of the measurement device. The signal emitted during the deformation of a mechanoluminescent material can be diverted to pre-existing scientific equipment with an optical fiber. During testing a 16-bit CMOS camera was used to determine strain distribution. Due to the highly elevated cost of such a system and the lack of spectral resolution, a considerably cheaper alternative is discussed in previous work [5]. Hamamatsu C12880MA spectrometer chip provides the necessary spectral and time resolution for mechanoluminescence measurement. In terms of mounting, the camera, when coupled with an appropriate lens, becomes quite heavy. As the intention is to explore untraditional configurations, during validation a height adjustable table was used, while in our studies finger clamp holder was used. Mentioned setup is shown in Fig. 4. A lens with a far focal point was used to facilitate the use of optical filters, that are easy to change due to the large gap between the 3D printed adapters for the camera and lens. The built-in 20 dB amplifier of the CMOS camera is capable enough to acquire an image even while using a bandpass optical filter.

## Design files

### Electronics

The device's electrical wiring schematic is shown in Fig. 7. The device consists of a Mean Well power supply, NHduino UNO and Arduino NANO (interchangeable with products developed by any other microcontroller distributor), DYLF-102 load cell, DY510 voltage stabilizer and amplifier (optional), Nema 23 23HS9430 stepper motor coupled with DM542A controller (smaller/larger stepper motors will require a different controller) and a CMOS BFLY-U3-23S6N camera (replaceable by the previously mentioned spectrometer or any other spectroscopic system).

**Fig. 5:**
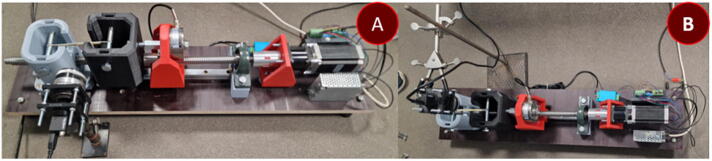
. (a) Tension configuration measured from the side and (b) overhead.

**Fig. 6:**
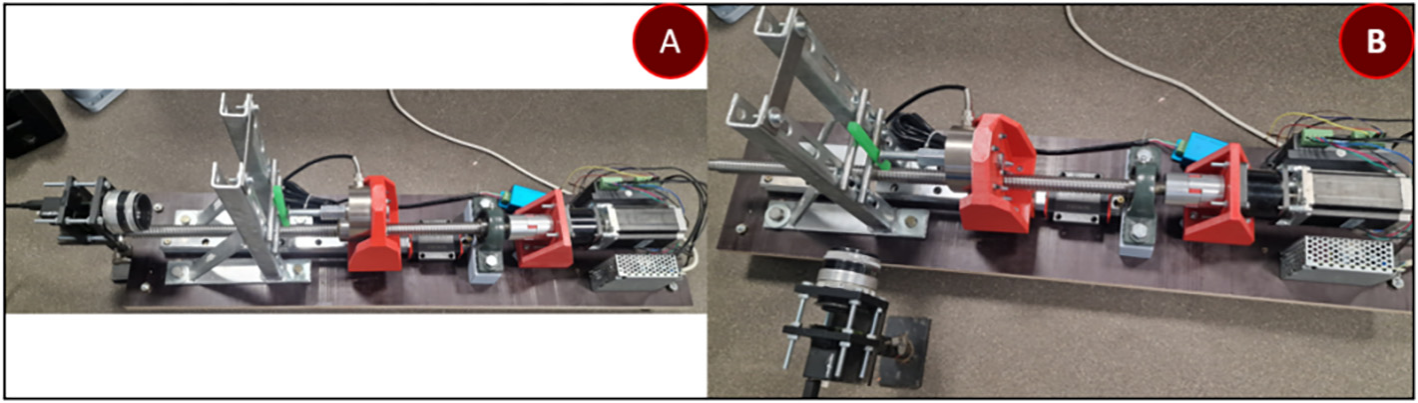
(a) Bending configuration measured from the side and (b) overhead.

**Fig. 7:**
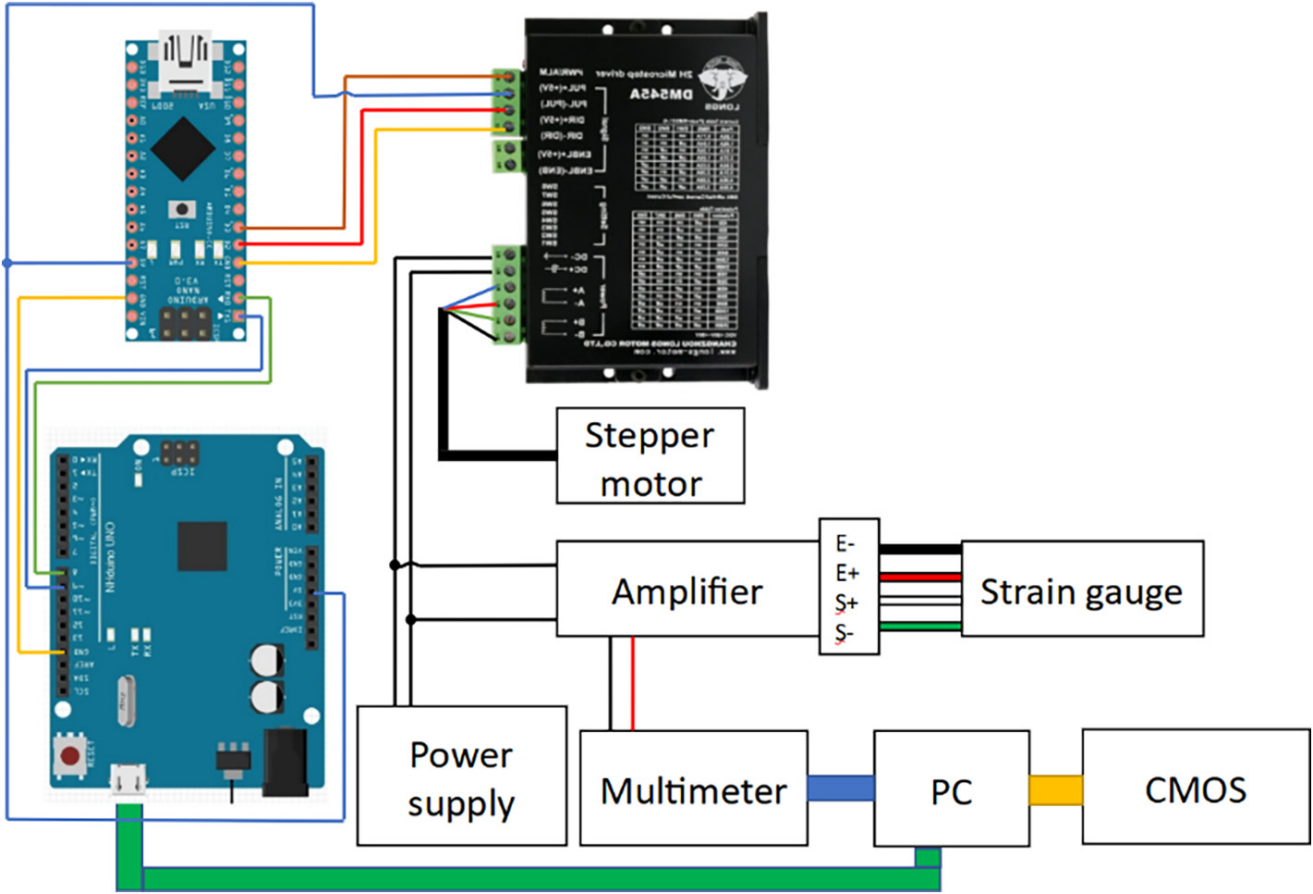
Electrical wiring of the material testing device.

### 3D printing

The 3D models were created with Autodesk Fusion 360 software and were sliced with Ultimaker Cura 4.8.0. Parts were 3D printed using Creality CR-10S Pro with a 0.4 mm nozzle installed. All models utilized a printing speed of 50.0 mm/s and a nozzle temperature of 210 ℃. The tensile testing configuration consists of 4 3D printed parts while a three-point flexural test consists of 2 parts that are shared between configurations. Part designs are optimized for fused filament fabrication while being mindful of structural integrity – regular PLA material was used.

The dimension of stepper motor support is 80.0 × 91.1 × 91.1 mm (Part 1) and separate 33 mm high supports are required in this prototype design to ensure proper alignment which does not have to be 3D printed. Separate supports will not be experiencing large pressure during measurements. Part 1 should be printed with at least 25 % infill with a layer height of 0.2 mm, which would take approx. 7 h and 24 min and 119 g of filament to print. The dimension of the load cell mount (Part 2) is 110.0 × 83.0 × 134.0 mm. Considerable force is to be expected upon this component and an infill of 75 % or higher is recommended. The prototype uses a part with 85 % infill which took 1 d and 32 min to print and 455 g of filament.

Parts 3 and 4 are required for tensile testing. Most of the pressure is exerted upon these parts which should be created with the highest structural integrity achievable. During testing parts with 85 % infill were used. The dimension of the mounts are 116.0 × 108.0 × 133.0 mm and 128.8 × 116.0 × 128.0 mm respectively which in turn took 1 d 21 h (871 g) and 1 d 18 h (809 g) to print. It must be noted that the static mount failed when around 500 kg of pull force was exerted upon the system. A slightly improved design for the moving mount (Part 3) is already included, but if higher forces are expected, both parts should be redesigned with screws as reinforcement along the Z-axis or manufactured entirely from metal.

### Software

The device is controllable in multiple ways. Microcontroller codes are provided in a text format and must be compiled and uploaded to both controllers by using the Arduino IDE environment. The Stepper motor moves the set number of steps upon receiving a singular impulse from the microcontroller. A master writer/slave receiver setup was implemented. If the intended application does not require communication during movement a single microcontroller would suffice; however, given the cost and availability, two separate controllers are preferred. Serial communication is used between microcontrollers and the computer. It is possible to control the systems through any software that provides the possibility to write to the serial buffer including Arduino IDE. Notes are provided within the.ino files (Arduino source code master, Arduino source code slave, Arduino source spectrometer chip). The prototype system was controlled with NI LabVIEW 12 (LabVIEW source code for CMOS, LabVIEW source code for spectrometer chip) for which compiled software executables (.exe files) are provided which in turn require LabVIEW Run-Time Engine 8 or later to run. Signal registration can be performed with either LabVIEW or the software provided by the camera manufacturer, or in the case of a spectrometer chip the software described in the article [5]. The signal from the load cell was digitalized by a programmable multimeter HM8012. The internal analog to digital converter of Arduino UNO can be used which would severely reduce the resolution of the load cell and eliminate the possibility to detect strain in the opposite direction because Arduino is capable of mapping 0–5 V to 0–1024 and the load cell emits −10–10 V (a voltage divider would be necessary).

## Design files summary

**Table:** 

**Design file name**	**File type**	**Open-source license**	**Location of the file**
Part 1	.stl file	CC BY 4.0	available at https://DOI.org/10.17605/OSF.IO/S4PG7
Part 2	.stl file	CC BY 4.0	available at https://DOI.org/10.17605/OSF.IO/S4PG7
Part 3	.stl file	CC BY 4.0	available at https://DOI.org/10.17605/OSF.IO/S4PG7
Part 4	.stl file	CC BY 4.0	available at https://DOI.org/10.17605/OSF.IO/S4PG7
Arduino source code master	.ino file	CC BY 4.0	available at https://DOI.org/10.17605/OSF.IO/S4PG7
Arduino source code slave	.ino file	CC BY 4.0	available at https://DOI.org/10.17605/OSF.IO/S4PG7
Arduino source spectrometer chip	.ino file	CC BY 4.0	available at https://DOI.org/10.17605/OSF.IO/S4PG7
LabVIEW project for CMOS	.zip file	CC BY 4.0	available at https://DOI.org/10.17605/OSF.IO/S4PG7
LabVIEW project for spectrometer chip	.zip file	CC BY 4.0	available at https://DOI.org/10.17605/OSF.IO/S4PG7
LabVIEW executable for CMOS	.zip file	CC BY 4.0	available at https://DOI.org/10.17605/OSF.IO/S4PG7
LabVIEW executable for spectrometer chip	.zip file	CC BY 4.0	available at https://DOI.org/10.17605/OSF.IO/S4PG7
Tensile testing configuration model	.stl file	CC BY 4.0	available at https://DOI.org/10.17605/OSF.IO/S4PG7
Three-point flexural test configuration model	.stl file	CC BY 4.0	available at https://DOI.org/10.17605/OSF.IO/S4PG7

## Bill of materials summary

**Table:** 

**Designator**	**Component**	**Number**	**Cost per unit -currency**	**Total cost -currency**	**Source of materials**	**Material type**
Arduino UNO	Arduino master	1	22.00 €	22.00 €	Arduino	Electronics
Arduino NANO	Arduino Slave	1	24.00 €	24.00 €	Arduino	Electronics
Load cell	DYLF-102	1	84.79 €	84.79 €	Amazon	Electronics
Voltage stabilizer and amplifier	DY510	1	50.69 €	50.69 €	Amazon	Electronics
Stepper motor	NEMA 23 23HS9430	1	37.99 €	37.99 €	Amazon	Electronics
Stepper motor controller	DM542A	1	19.99 €	19.99 €	Amazon	Electronics
Power supply	Rd-125–1224	1	28.84 €	28.84 €	Local hardware store	Electronics
Spectrometer chip	C12880MA Spectrometer	1	190.00 €	190.00 €	Hamamatsu	Electronics
Blackfly S USB3	BFLY-U3-23S6M-C	1	519.00 €	519.00 €	Teledyne FLIR	Electronics
Planetary gear set	Nema23 planetary gearhead 1:10	1	73.00 €	73.00 €	CNCdrive	Metal
Rail	HGR 25 linear rail	1	4.20 €	4.20 €	CNCdrive	Metal
Carts	HGW 25 linear slide	2	23.00 €	46.00 €	CNCdrive	Metal
Lead screw	SFU1204 ball screw	1	3.00 €	3.00 €	CNCdrive	Metal
Lead screw nut	SFU1605 - ball nut	1	30.00 €	30.00 €	CNCdrive	Metal
Lead screw bearing	UPC201 bearing support with a self-centering bearing	1	6.00 €	6.00 €	CNCdrive	Metal
Connector	8 mm to 12 mm rigid coupling	1	1.41 €	1.41 €	Local building materials	Metal
Screws	Wood screws 3.9x38	6	0.02 €	0.12 €	Local building materials	Metal
Bolts	M5x16 bolts	18	0.04 €	0.72 €	Local building materials	Metal
Bolts	M4x20 bolts	12	0.03 €	0.36 €	Local building materials	Metal
Bolts	M12x100	2	1.12 €	2.24 €	Local building materials	Metal
Nuts	M5 nuts	18	0.01 €	0.18 €	Local building materials	Metal
Nuts	M4 nuts	12	0.01 €	0.12 €	Local building materials	Metal
Nuts	M12	2	0.18 €	0.36 €	Local building materials	Metal
Metal bracket	Mounting bracket 500 mm	2	5.51 €	11.02 €	Local building materials	Metal
Part 1	3D printed support	1	2.87 €	2.87 €	Pro-mix	3D printing
Part 2	3D printed support	1	10.96 €	10.96 €	Pro-mix	3D printing
Part 3	3D printed support	1	20.98 €	20.98 €	Pro-mix	3D printing
Part 4	3D printed support	1	19.48 €	19.48 €	Pro-mix	3D printing
Base plate	Plywood	1	6.20 €	6.20 €	Local building materials	Wood

## Build instructions

Provided diagrams are not to scale and features are exaggerated.

1. Cut the plywood to size (820 × 170 mm);
2. Mark a center line along the length of the board;
3. Align the rail with the drawn line through the screw holes on the rail;
4. Mount with wood screws whilst leaving the last holes empty on both ends of the rail;
5. In the hole furthest from the end of the board screw in a screw until it sits at least 5 mm above the rail to stop carts from slipping off the rail;
6. Carefully transfer both carts to the rail and fill the last hole in the rail. IMPORTANT! If carts manage to derail or the transfer is unsuccessful, spring-loaded ball bearings will eject and it is extremely challenging to reassemble them;
7. Create a 50.5 mm high spacer for lead screw bearing either by 3D printing it or from the plywood cut-outs with M20 bolt holes, lead screw bearing can be used for alignment;
8. Mount the lead screw bearing perpendicular and centered to the rail, and disregard any misalignment with the sides of the board. If the rail is misaligned with the screw bearing, then the lead screw will bend during measurements which will lead to excess stress upon the parts;
9. Mount the 3D parts to the carts for the chosen type of deformation (Fig. 1 or Fig. 2). In the case of the three-point flexural test, mount the metal brackets to the end of the board. The three-point flexural test requires a singular cart, but due to the challenging nature of removing a cart from the rail without losing the ball bearing, it is advised to leave the spare cart on the rail for future use if already installed;
10. Thread lead screw through the parts and seat it in the bearing;
11. Fasten the lead screw nut to part 2;
12. Join the connector to the lead screw after the bearing;
13. Assemble the stepper motor and planetary gear-set. IMPORTANT! Do not create tension between the motor shaft and gear set. It will create friction between the shaft and the first gear head, leading to excessive wear and premature failure;
14. Mount assembled system to part 1;
15. Create a 33 mm high spacer for part 1;
16. Join part 1 with a previously mounted connector;
17. Mount part 1 to the board. It is done after joining with the connector to retain alignment with the rail;
18. Inspect the system. While manually turning the connector, either way, part 1 should easily slide along the rail and the motor shaft should spin. If the cart is not sliding, try lubricating the system and checking the alignment. If the motor shaft is static, disassemble and reassemble the planetary gear-set and tighten the mounting clamp harder as it is prone to slipping;
19. Do the necessary wiring as shown in Fig. 7;
20. Set the SW pins of the stepper motor controller to the desired speed by referencing the label on it or by following the manual provided by the manufacturer.

## Operation instructions

### To use the device with the provided LabVIEW program

Install LabVIEW RTE 8 or later and LabVIEW VISA, which is required to use hardware interface for communication.

Check if the system is wired as shown in Fig. 7.

**Fig. 8:**
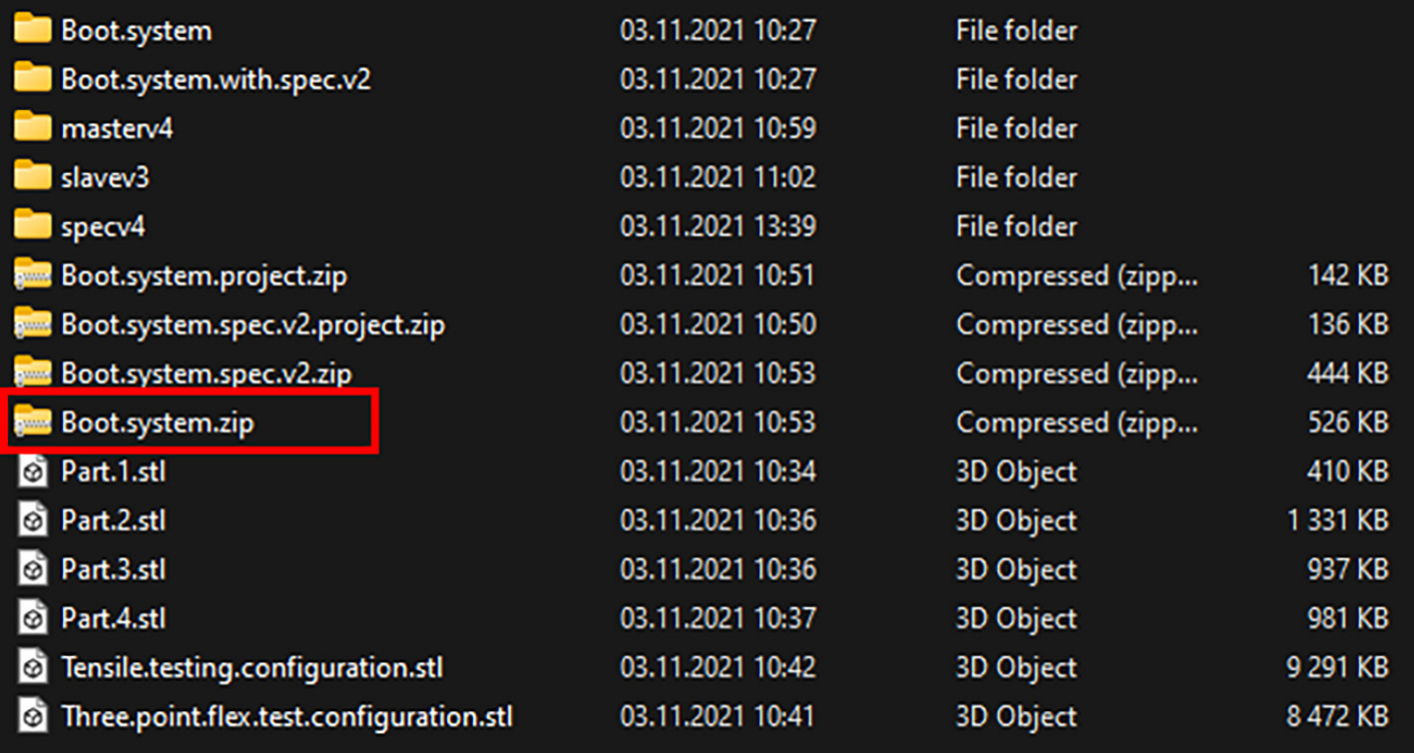
Compressed file containing the program executable.

Extract the file labeled Boot.system.zip (Fig. 8) to a known location.

**Fig. 9:**
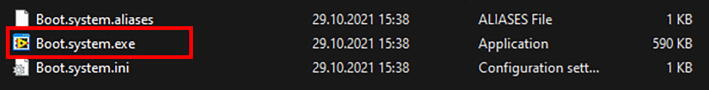
Label of the executable file.

Run the file labeled Boot.system.exe shown in Fig. 9 which has to be within the same folder as the.aliases and.ini files.

**Fig. 10:**
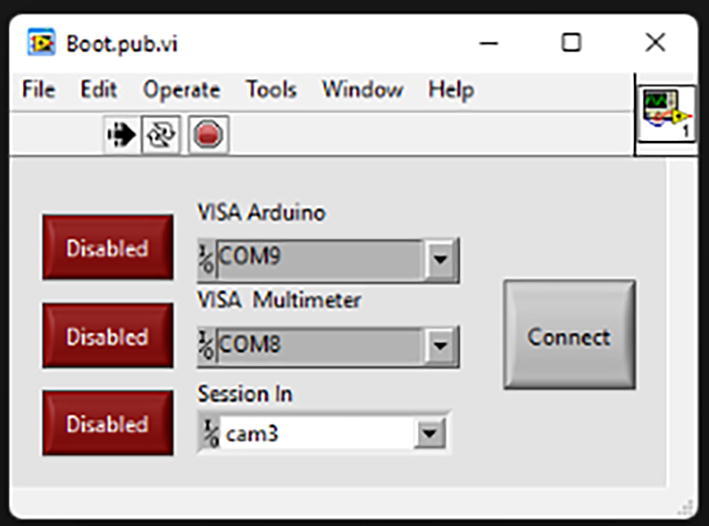
A pop-up window asking to specify the connected ports.

After running the program, you will be asked to specify communication ports for the microcontroller responsible for controlling the stepper motor, programmable multimeter, and the virtual session of the camera or the microcontroller responsible for the spectrometer chip (Fig. 10). Toggle the state of the connected apparatus to Enabled and click Connect.

**Fig. 11:**
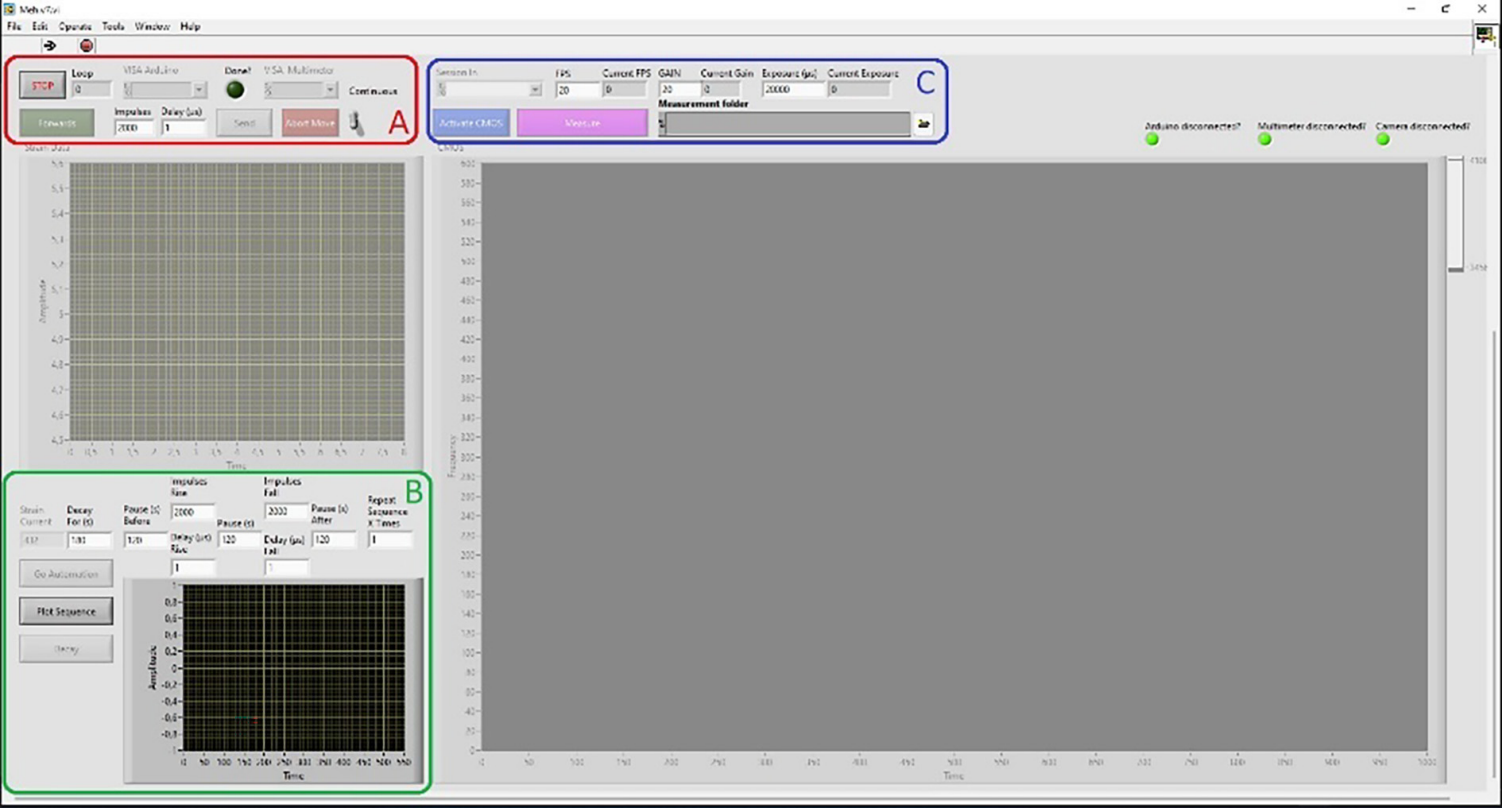
The main interface of the program.

A separate pop-up window will arise which contains all the relevant configurable (Fig. 11). Any content related to a disabled component will be greyed out and non-interactable.

Part (A) of the LabView program will display its current logical cycle if all movements are done, allowing to change the current movement direction and the number of impulses to send in said direction with the respective delay between impulses which directly controls the speed of movement. The switch labeled “Continuous” will resend the direction, impulses, and delay command upon receiving a “Done” signal from the master Arduino until switched off. “Abort Move” will stop all movement by triggering a system interrupt and sending a blank command to the slave Arduino.

Part (B) is responsible for the automatization of mechanoluminescence measurement. Button “Decay” performs a background measurement for a set number of seconds without deformation and each spectrum/image is saved in the folder specified in part (C). The plot sequence serves as a purely illustrative tool and “Go Automation” will respect the current direction selected. Acquired images will be automatically saved which does continue during all three pause periods.

Part (C) contains the configurable relevant to a CMOS camera. Activate starts the acquisition cycle, shows the last image, and does not save it. It is possible to set the frame rate, gain, and exposure time. Automatic adjustment of exposure and frame rate is permanently disabled, and the data format of images is set to 16-bit.

**Fig. 12:**
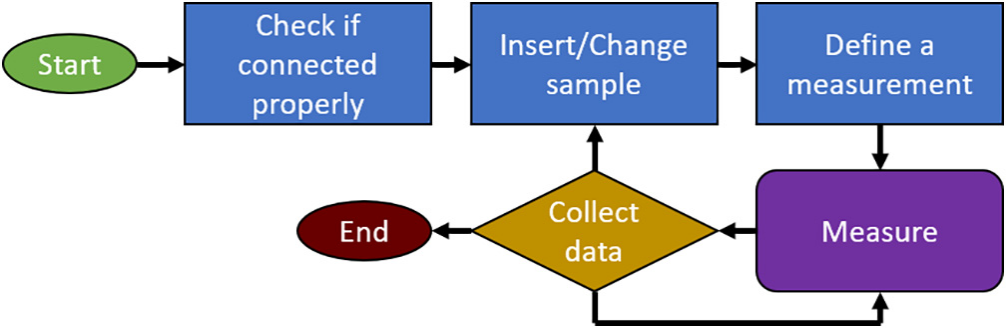
Block diagram of the measurement procedure.

The measurement procedure should follow the block diagram shown in Fig. 12.

Provided program for a spectrometer chip system looks and works the same way.

### To use the device without the provided program

The chosen CMOS system is not directly adaptable and usable by open-source alternatives. The drivers provided by the manufacturer lack a detailed description of the acceptable variables and the output returned by the functions that themselves are not named in other words the dynamic-link library lacks a header file. It is required to use the manufacturer's software or find compactable third-party drivers. In this system, a proprietary LabVIEW driver was installed. A spectrometer chip system is advisable for the creation of open-source alternatives. It is possible to link the spectrometer chip to the master Arduino, the provided additional code should be uncommented and the delay set to a value of at least 200 ms, otherwise, the outbound data will allocate most of the internal memory responsible for data transfer of the master Arduino and the chip will cease communication until emptied by the receiver or discarded after a timeout, the serial buffer will overflow.

For material testing purposes it is enough to link the multimeter to a computer and to save the reported voltage change during measurements.

## Validation and characterization

To properly test the created system a well-known mechanoluminescent material had to be chosen. Strontium aluminate and epoxy resin composite were chosen due to their being extensively researched currently [6], [7], [8], [9], [10], [11], [12].

Currently, it is not completely clear as to why certain materials exhibit mechanoluminescent properties, luminescence is observed during rubbing or breaking, or the afterglow intensity changes during deformation like bending or compression. It has been mainly accepted that SrAl_2_O_4_:Eu^+2^, Dy^+3^ is mechanoluminescent due to piezoelectrically induced detrapping, which arises from its crystalline structure. During deformation, the lattice deforms very slightly which in turn creates a small piezoelectric field. Due to the presence of uncompensated internal field atoms are forced to slightly rearrange which might be enough to release previously captured charge carriers from lattice imperfections, leading to light emission [4]. Generally, increased light emission is observed during deformation while a sample is being bent and while it returns to its initial position. Epoxy resin is used as a binder due to strontium aluminate being a powder. It serves no purposes other than shaping the powdered substance and transferring mechanical strain to particles within it. Multiple kinds of binders can be used for sample preparation if their mechanical properties are comparable to strontium and are transparent to ultra-violet (required for excitation) and visible light (otherwise luminescence will be absorbed) [13].

The presented device is intended for spectroscopic applications, that is for the study of light emission during elastic deformation. Plastic deformation or in the worst case fracture of a sample is a costly and time-consuming endeavor. The presented system is designed to repeatedly deform a sample by a few millimeters with high precision. It is not designed for an accurate study of material properties. Initially, the system was used in a tensile testing configuration to study composite coatings on alumina 6082 plates as they have a higher tensile strength than shear strength. This is the only configuration, that experienced a structural failure that was not done intentionally. Part 4 failed at around 650 kg of force and the nut inserts served as a weak point of the design. Although unadvised, the system should be capable of studying material properties up to 300 kg and could be adapted for classical material testing by reinforcing printed parts 2, 3, and 4 with steel bars spanning the width and the height of the structure.

**Fig. 13:**
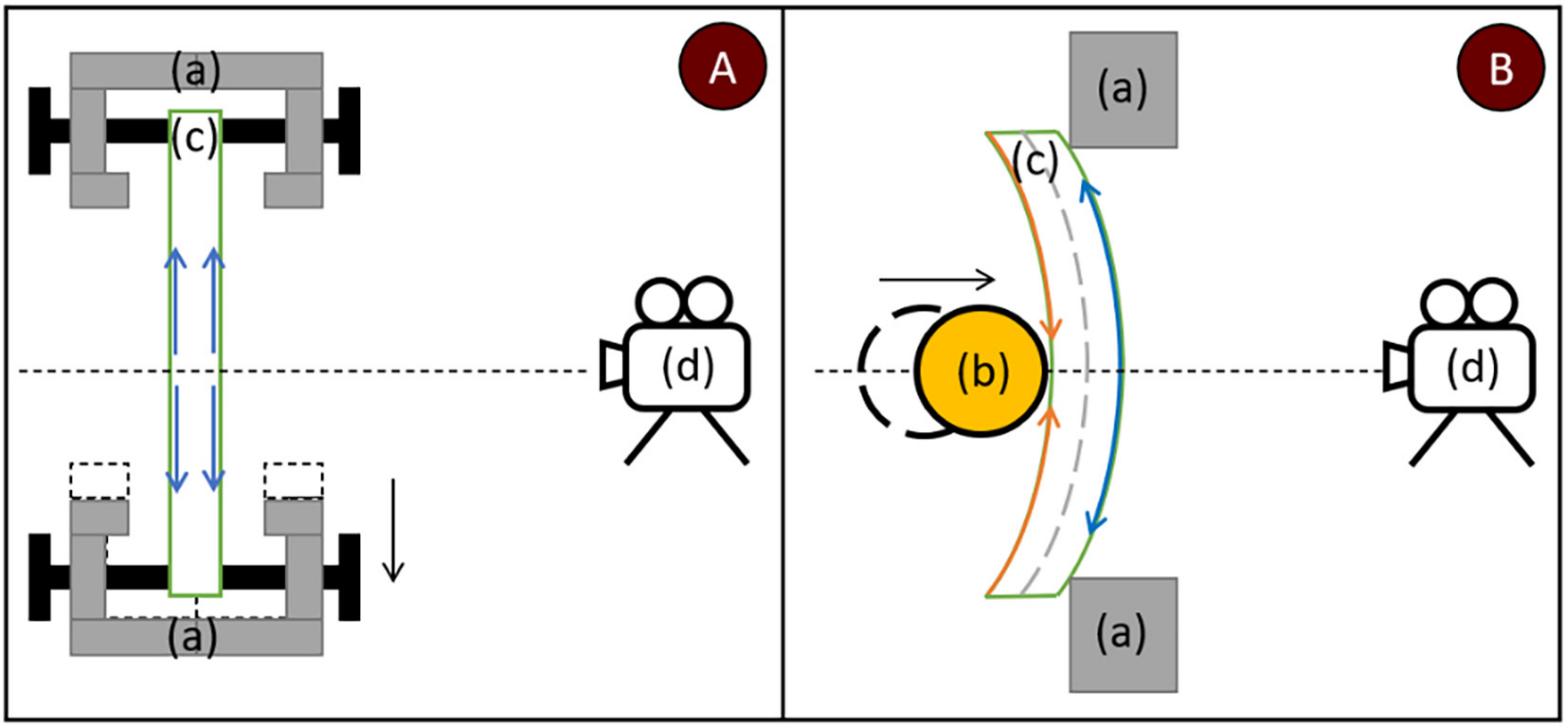
Experimental scheme for (A) tensile testing and (B) three-point flexural test. (a) Supports, (b) deforming object for three-point flexural test configuration, (c) sample, and (d) camera. Black arrows represent the direction of movement, orange arrows represent compression and blue elongation. (For interpretation of the references to colour in this figure legend, the reader is referred to the web version of this article.)

A top-down view of the experimental scheme is shown in Fig. 13. The tensile testing configuration would provide a homogeneous strain distribution, and the sample would not move in relation to the signal acquisition device if outfitted with a more reproducible sample clamping mechanisms like clamping jaws or gimballed mounts. As this device is intended for mechanoluminescence measurements, a homogeneous strain distribution is undesirable and creates a hurdle, that can be avoided by creating samples with irregular cross-sections. In short, tensile testing configuration complicates sample creation but simplifies data processing. Three-point flexural test configuration provides 2 major advantages and a singular disadvantage. Generally, binders tend to be ductile and are easily bendable but quickly fracture under extensive elongation without support, which during bending is provided by inner layers. While a sample is being bent, the strain distribution forms a gradient from the center outwards for samples with a regular cross-section and can be further modified with ease. Since the overall geometry changes quite drastically, it must be accounted for during data analysis due to the sample being brought towards or away from the signal acquisition equipment. The magnitude of the error due to geometry change is shape and deformation amplitude-dependent, which during validation was estimated to be no more than 2 %.

**Fig. 14:**
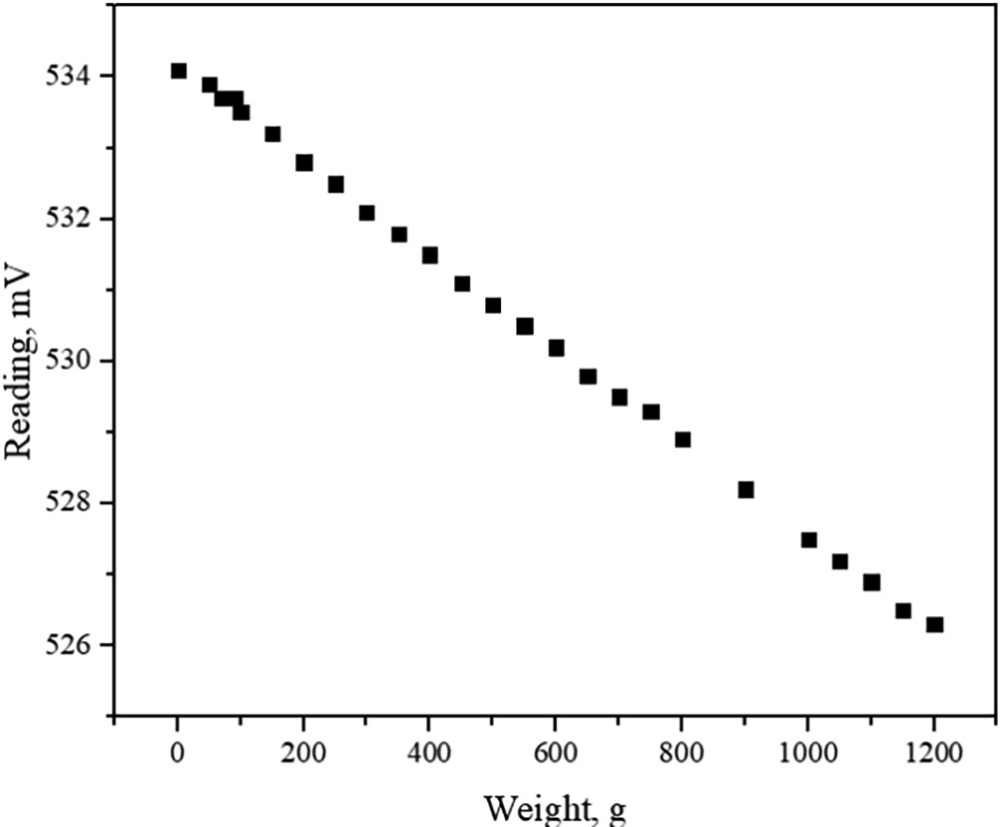
Load cell calibration with weight set.

The performance and accuracy of the spectrometer chip were extensively characterized in previous work [5]. The only limitations of a CMOS system are the data cap of the USB connection, the clarity of the lens used, and the area of the aperture (during validation was not used). A single 16-bit image is close to 4 MB in size, the typical data transfer speed of USB 3.0 is around 75 MB/s, and it is not technically possible to continuously capture 40 fps with 16-bit pixel depth without a capture card. Image clarity and signal intensity are lens dependent and will improve with quality equipment that is not added to the bill of materials. The price of camera optics varies drastically, a cheap option can cost a few tens of dollars, while a quality photography grade lens can go up hundreds of dollars. The weight applied to the sample was measured with (DYLF-102) a spokes type bidirectional load cell that measures pull or pressure up to 1000 kg. As we decided to digitalize load cell voltage with a programmable multimeter, the system had to be calibrated. In this prototype system, calibration was performed with a laboratory-grade weight set which is shown in Fig. 14. The dynamic range of load cell used is from −10 to 10 V with stability to ±0.001 V when paired with a voltage amplifier and stabilizer. It has to be noted, that in terms of strain measurements tensile testing configuration is more precise as the strain transfer shaft is supported at both ends firmly and produces up to ±0.005 V noise while the system is in motion, full theoretical stability is only achievable while the system is stationary. Three-point flexural test configuration produces a rather high noise of ±0.020 V while the deforming object is not pressing against the sample. During validation, a 6.50 cm long deforming object was used and as it was directly attached to the load cell, it wobbled and created noise. A shorter deforming object is advised for more precise strain measurements while performing three-point flexural tests. The weight measurement error created by said noise completely depends on the accuracy of calibration and the span set on the amplifier (if implemented). For mechanoluminescence measurements, the system was prestrained to reduce noise, as mechanoluminescence is observable when the sample changes shape and experiences a strain gradient. The maximal movement speed of the stepper motor, when paired with a planetary gear set, was determined to be 0.62 ± 0.008 (mm/s). By increasing the delay between impulses from 1 to 2 microseconds the speed decreases to 0.31 (mm/s).

**Fig. 15:**
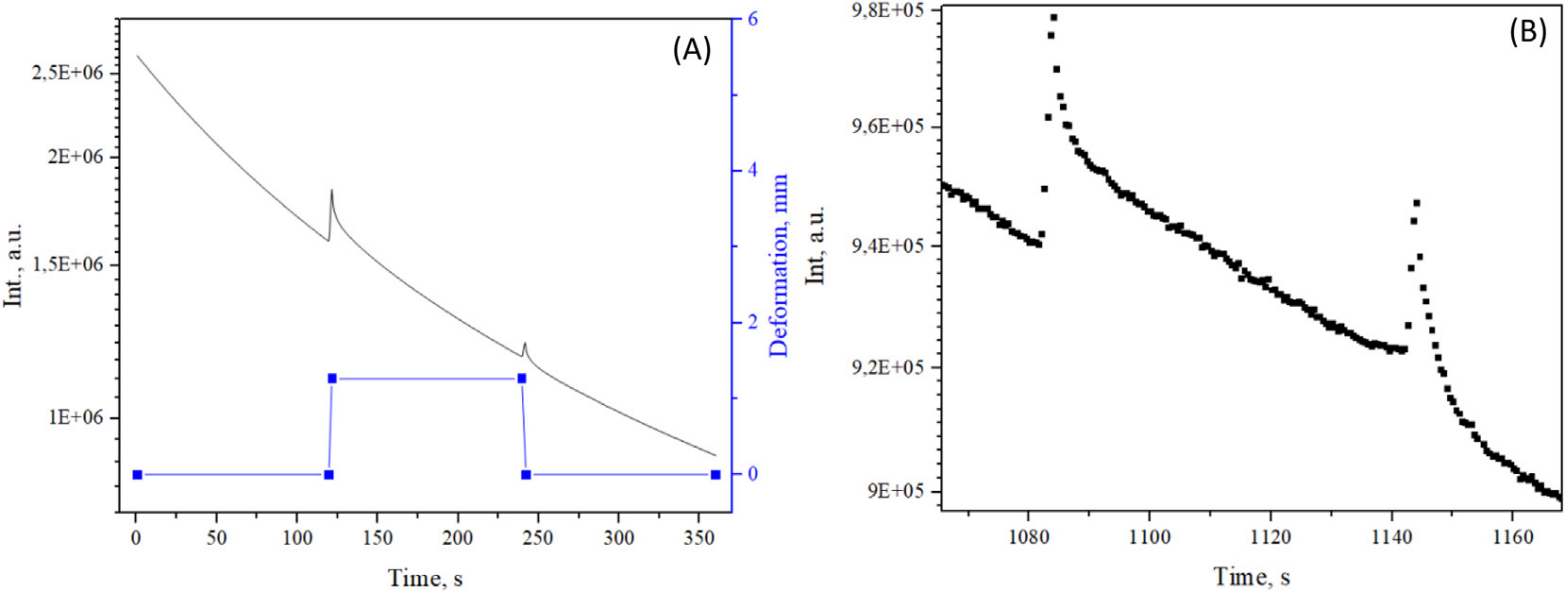
(A) Integral signal intensity changes of strontium alumina and epoxy sample during one cycle, (B) integral signal intensity after 10 deformation cycles.

Dysprosium and europium-activated strontium aluminate nanocrystals were mixed with epoxy resin in a syringe which is a cylindrical cavity. Measurements performed with a cylindrical sample and a three-point flexural test configuration are shown in Fig. 15. The sample was irradiated with a UV light source for 5 min. The first 5 min after shutoff was not registered due to the intensive afterglow of SrAl_2_O_4_:Eu^+2^, Dy^+3^ which leads to overexposure. After 2 min the sample was deformed by 2 mm, observed for 2 min, and relaxed afterward. During a single measurement, 10 cycles were performed. Mechanoluminescence was detectable after 10 cycles and 18 min after irradiation. This test was done with auto-gain functionality disabled and proves, that the dynamic range of the suggested CMOS camera is enough to study a sample while it is almost fully charged and almost depleted which is highly desirable for spectroscopic studies.

**Fig. 16:**
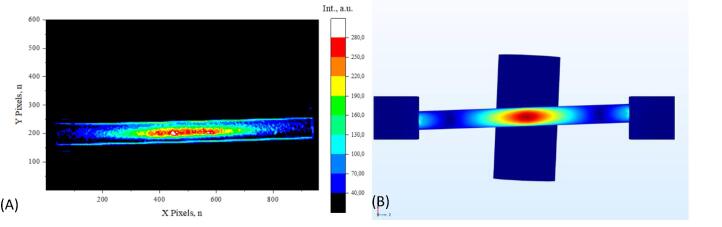
Validation of a fully circular sample: (A) measured signal intensity distribution, (B) theoretical strain distribution.

**Fig. 17:**
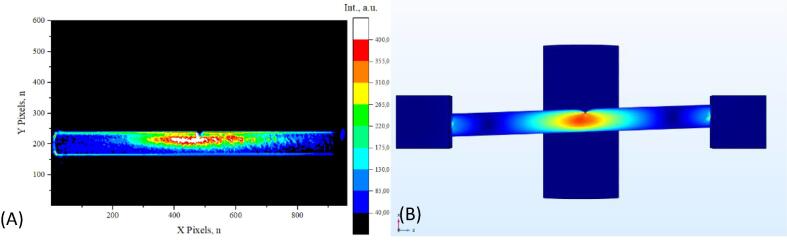
(A) Measured strain distribution, (B) Calculated strain distribution.

Detected strain distribution was compared to theoretical calculations which is one of the main purposes of the presented system. As previously mentioned, there is no concrete explanation of the mechanism that causes certain materials to emit light upon deformation. The purpose of this prototype system is to study asymmetric strain distributions and the intensity of mechanoluminescence under unconventional circumstances. For validation purposes, a sample with a fully circular cross-section was created and tested. The registered signal intensity distribution is shown if Fig. 16 (A) and the calculated strain distribution is shown in Fig. 16 (B). The sample is not clamped to the metal brackets which allows them to act as rollers or support pins. During theoretical calculations, the sample geometry is not connected to the square rigid domains. No other boundary condition is imposed upon the sample geometry, except movement downwards or upwards is forbidden. The theoretical simulation predicted symmetrical strain distribution which was observed during testing. Next, a small indent was made in the sample to make a starkly different strain distribution. Measured and calculated results for an indented sample are shown in Fig. 17. Theoretical simulation predicted a concentrated strain distribution at the tip of the indent and an overall thinner distribution along the surface of the sample. It was observed, that the mechanoluminescence registered from an indented sample has a lot higher intensity and the overall distribution is narrower. A small deviation from the theoretical model is to be expected. It is noticeable to the naked eye that the particle distribution in the test sample was not homogenous, while theoretical results are for samples with isotropic mechanical properties. For three-point flexural tests, it is hard to mitigate movement as previously mentioned. The camera is observing the sample at a slight angle and when the sample is deformed by 2 mm the center moves 2 pixels downwards. For validation purposes, it was ruled as a meaningless error and was not accounted for during data processing. Measured strain distribution is made clearer by calculating the intensity difference between a frame right before deformation and a frame with the highest measured mechanoluminescence. A different approach to strain visualization is provided in one of our publications [14].

**Fig. 18:**
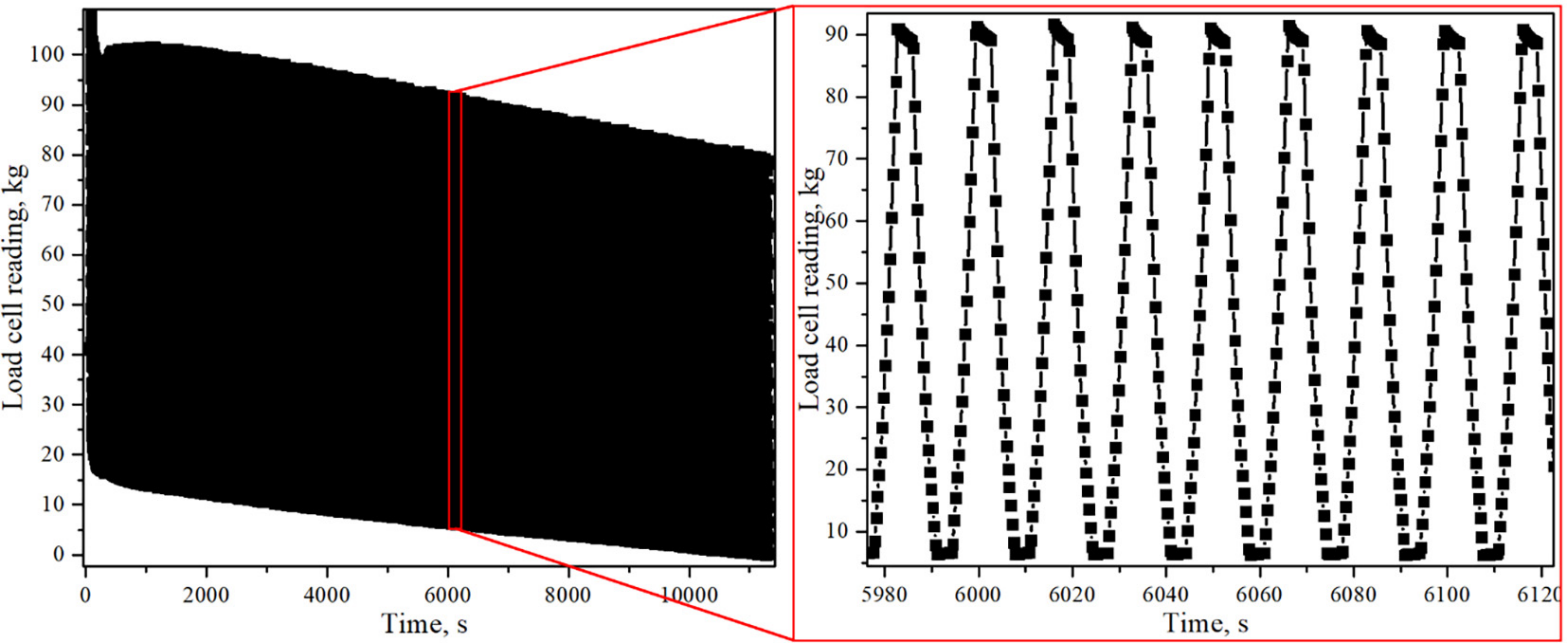
fatigue test results of a composite polymer sample.

Although the system is not intended for conventional material testing it is possible by fabricating appropriate rollers. For mechanoluminescence measurements, a negligible deviation from the convention is observed as the number of cycles rarely exceeds 30. For validation purposes, 9.6 mm in diameter and 10 cm long rollers with M3 screw size holes at both were fabricated, of which a glimpse can be seen in Fig. 6. Rollers were positioned 2.4 mm above and below the moving pin. A fatigue test was performed on an additively manufactured plastic composite sample that was 10 cm long, 16 mm wide and 6 mm thick. The sample was subject to 40 kg of initial stress and deformed by 0.5 cm in both directions 676 times before it succumbed to material fatigue, load cell readings gathered during testing are shown in Fig. 18.

## Conclusion

Mechanoluminescence measurements require specific equipment that is not currently commercially available. The described prototype is capable of testing polymer-particle composite coatings and cast shapes in which one dimension exceeds 9 cm without additional modifications. Capabilities (and limitations) of the constructed prototype are:

Capable of autonomous mechanoluminescence measurements up (but not limited to) 10 deformation cycles.

CMOS camera set-up is capable of measuring luminescence intensity with spatial resolution, the spectrometer chip exchanges spatial resolution for spectral distribution.

The use of a stepper motor facilitates high repeatability, and precision and is capable of exerting immense force upon the sample. Used Nema 23 23HS9430 has a torque of 3 N∙m and is coupled with a 1:10 planetary gearset and a leadscrew.

The system is highly modifiable due to the use of mostly 3D parted parts and is mainly Arduino controlled.

Achievable strain is mainly limited by the use of said 3D printed parts, catastrophic failure depends on the quality and composition of filament used and the part infill, as well as the quality of the printer itself. Poor layer adhesion and low infill will provide subpar results.

16-bit camera easily exceeds the bandwidth of a PC USB 3.0 interface when operated at 40 fps without pixel binning. Either the resolution or the frame rate has to be sacrificed to avoid dropping frames.

## Declaration of Competing Interest

The authors declare that they have no known competing financial interests or personal relationships that could have appeared to influence the work reported in this paper.
